# Peptide Nanoparticle-Mediated Combinatorial Delivery of Cancer-Related siRNAs for Synergistic Anti-Proliferative Activity in Triple Negative Breast Cancer Cells

**DOI:** 10.3390/ph14100957

**Published:** 2021-09-23

**Authors:** Anna Egorova, Ivan Pyankov, Marianna Maretina, Vladislav Baranov, Anton Kiselev

**Affiliations:** 1 Department of Genomic Medicine, D.O. Ott Research Institute of Obstetrics, Gynecology and Reproductology, Mendeleevskaya Line 3, 199034 Saint Petersburg, Russia; egorova_anna@yahoo.com (A.E.); marianna0204@gmail.com (M.M.); baranov@vb2475.spb.edu (V.B.); 2 Department of Genetics and Biotechnology, Saint Petersburg State University, Universitetskaya Emb. 7/9, 199034 Saint Petersburg, Russia; vanypyankov@gmail.com; 3 Institute of Chemistry, Saint Petersburg State University, Universitetskii Pr. 26, 198504 Peterhoff, Russia

**Keywords:** triple negative breast cancer, RNAi, peptide nanoparticles, AQP3, CDC20, COL4A2

## Abstract

Triple negative breast cancer (TNBC) is one of the deadliest types of cancer for women of different age groups. Frequently this cancer does not respond to conservative treatment. Combinatorial RNAi can be suggested as an advanced approach to TNBC therapy. Due to the fact that TNBC cells overexpress chemokine receptor 4 we used modular L1 peptide-based nanoparticles modified with CXCR4 ligand for combinatorial delivery of siRNAs suppressing major transduction pathways. TNBC cell line MDA-MB-231 was used as a cellular model. Genes encoding the AQP3, CDC20, and COL4A2 proteins responsible for proliferative activity in TNBC cells were selected as RNAi targets. The siRNA binding ability of the carrier was studied at different charge ratios. The silencing specificity was demonstrated for all siRNAs studied. Alamar Blue proliferation assay has shown significant reduction in the anti-proliferative activity after combinatorial siRNA transfection compared to single siRNA delivery. The most significant synergistic effect has been demonstrated for combinatorial transfection of anti-COL4A2 and anti-CDC20 siRNAs what resulted in 1.5–2 fold inhibition of proliferation and migration of TNBC cells. Based on our findings, we have concluded that combinatorial treatment by CXCR4-ligand modified L1-polyplexes formed with AQP3, CDC20, and COL4A2 siRNAs effectively inhibits proliferation of TNBC cells and can be suggested as useful tool for RNAi-mediated cancer therapy.

## 1. Introduction

The mammary gland/breast is a hormone-dependent organ of the female reproductive system [1]. Mammary cancer is one of the most common types of cancers among women of any age, and for 30 years the number of detected breast cancer cases has increased by more than 40% [2]. Some successes were achieved in the mammary tumors treatment by negatively affecting estrogen (ER) and progesterone (PR) receptors as well as human epidermal growth factor receptor 2 (HER-2) when breast cancers displayed these receptors [3,4,5]. However, chemotherapy used in the mammary cancer treatment has a gonadotoxic effect and leads to a decrease or complete loss of fertility in women of reproductive age. It is worth noting that the triple negative breast cancer (TNBC) is characterized by the lack of ER- and PR-, as well as HER-2 gene expression [6]. Due to the absence of targeted therapy, TNBC remains a more aggressive among all mammary cancer subtypes. The lack of proper response to the chemotherapy results in the high recurrence rate and as a consequence short overall TNBC patients’ survival when compared to hormone-positive breast cancers [7,8]. TNBC is characterized by genomic and molecular aberrations that lead to dysregulation of signaling pathways [9,10]. A possible way for effective and targeted as well as fertility-preserving treatment of TNBC may be suppression of the signal transduction pathways or related proteins with the subsequent inhibition of the cancer cell proliferation and migration; this, in turn, can reduce the metastases.

TNBC progression and development is regulated by several pathways, including NF-kB, JAK/STAT3, PI3K–AKT–mTOR and PI3K/AKT, however only few of them are found to be useful for targeted therapy [11,12,13,14]. To address this issue an advanced therapy should be explored using novel approaches and/or a combination of already established strategies. Advanced technology such as RNAi in the combination with chemotherapy could be a promising approach to treat TNBC but some obstacles are present such as efficiency and specificity of siRNA delivery. The discovered phenomenon of RNA interference (RNAi) makes it possible to relatively easy silence the corresponding gene expression [15]. RNAi could become the targeted therapy for TNBC in order to downregulate the certain genes involved in the tumor development and progression. Previously it was shown, thatcancer cell proliferation, migration and metastasis are regulated by SDF-1 (stromal cell derived factor 1)/CXCR4 axis which stimulate downstream G-protein signaling leading to activation of protein kinase cascades [16]. CXCR4 receptor is abundantly overexpressed on surface of TNBC cells and can be used as specific target for siRNA therapeutics delivery [17,18]. Also, SDF-1-induced breast cancer signaling and migration requires aquaporin-3 (AQP3), which is a member of the aquaporin water channel family functioning as water and glycerol transporter [19]. It is also required for directional mammary cancer cell migration via SDF-1-induced cellular uptake of hydrogen peroxide [20]. AQP3 also may be contributed to ATP generation energizing cancer cells to proliferate and develop tumor [21]. AQP3 gene silencing was shown to significantly reduce proliferation and migration of TNBC cells [22]. An important cancer feature is the upregulation of cell division, due to the activation of proteins involved in cell cycle. One of these proteins is the cell division cycle protein 20 (CDC20), a participator of multiple cell cycle regulatory pathways that required for progression and completion of mitosis [23]. It activates a complex of proteins that regulate the anaphase promoting complex (APC), which initiate chromatid separation and the start of cell division at anaphase [24]. Thus, CDC20 suppression can be resulted to the arrest of the cell cycle and thereby reduction of the tumor progression [25]. CDC20 is highly expressed exactly in TNBC cells but not in hormonal breast cancer cell lines [26]. CDC20 downregulation in TNBC cells was shown to result in 40% inhibition of cell growth [27]. Another promising target for the breast cancer treatment is the proteins of the basement membrane components. One of such proteins is collagen type 4 alpha-2 (COL4A2), which is extremely important for functioning and stability of vascular basement membrane [28]. COL4A2 was demonstrated to be involved in the tumorigenesis of the reproductive system organs, prostate cancer, epithelial ovarian cancer, uterine leiomyoma, breast cancer and etc. [29,30,31,32]. It was also shown that COL4A2 is overexpressed in TNBC cells; therefore, it can be assumed that the COL4A2 protein plays an important role in the pathogenesis of this breast cancer subtype [33]. Suppression of COL4A2 gene in TNBC cells led to significant reduction in cell proliferation and migration level [34].

For RNAi to perform effectively, siRNA needs to be compacted with a vehicle to prevent extracellular degradation and to ensure its release to the cytoplasm [35]. To achieve targeted delivery to tumor cells we previously developed a new ligand peptide derived from N-terminus of chemokine SDF-1 for CXCR4 receptor specific binding and include it in the modular peptide vehicle [36,37,38]. The resulting L1 carrier was effective in siRNA delivery in vitro and in vivo studies [39,40]. Also, we demonstrated a 60–80% silencing of GFP expression after treatment of the triple negative breast cancer MDA-MB-231 cells with L1/siRNA complexes [41].

Here, a combinatorial approach based on CXCR4-specific delivery of TNBC-related siRNAs was studied. We used siRNA/L1 complexes containing siRNA against AQP3, CDC20 and COL4A2 for targeted inhibition of TNBC cell proliferation and migration. We analyzed the anti-tumor effects of AQP3, CDC20 and COL4A2 targeting alone and in combinations. Synergistic anti-tumor effects after combinatorial siRNA delivery were studied. We identified a more effective combination of siRNAs for therapeutic silencing that may provide a basis for targeted TNBC gene therapy.

## 2. Results and Discussion

The pharmaceutical attractiveness of siRNA for tumor gene therapy is based on its properties such as temporary effect due to lack of genome integration and low variation of chemicals because of controlled synthesis [42]. The development of siRNA-based cancer therapy has greatly relied on the effect of increased tumor permeability and retention [43]. The identification of specific target genes for suppression in TNBC opens up new possibilities for siRNA-based therapy of the disease. It could be interesting to study how the combinatorial silencing of the factors specific for TNBC can influence such important phases for the tumor progression as cell proliferation and migration.

### 2.1. In Vitro Transfection of MDA-MB-231 Cells

**Figure 1:**
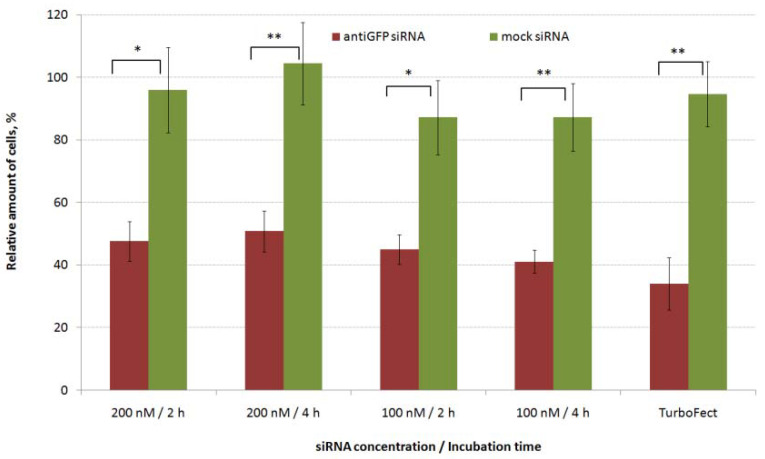
Silencing of *gfp* expression after treatment of MDA-MB-231 cells with L1/antiGFP siRNA complexes at 8 to 1 of N/P ratio. * *p* < 0.05, ** *p* < 0.01 when compared with cells treated by mock siRNA-complexes.

MDA-MB-231 breast cancer cells with constant gfp gene expression were used for in vitro transfection by L1/anti-GFP siRNA complexes (Figure 1). CXCR4 surface expression is known to be upregulated in TNBC cells and is an independent prognostic factor for disease relapse [44], so the L1 peptide carrier containing the ligand to CXCR4 receptor is a suitable vehicle for the marker and therapeutic siRNAs delivery into the cells. It should be noted that proliferation studies require low density to prevent contact inhibition of cell growth and thus require shorter time of incubation of cells with siRNA-polyplexes. On other hand, standard migration protocols employ high cell density and 4 h incubation [39]. Previously, effective inhibition of gfp expression was registered for MDA-MB-231 cells seeded in low density after their 2.5 h incubation with siRNA-polyplexes [41]. In order to harmonize proliferation and migration protocols we optimized L1-polyplexes mediated transfection: the siRNA-complexes were left for 2 and 4 h in a transfection medium and two siRNA concentrations were tested. As shown in Figure 1, control mock siRNA-containing polyplexes did not demonstrate gfp gene expression silencing, whereas, L1/anti-GFP-siRNA complexes delivery resulted in significant decrease in gfp expression up to 40–50%.

These data were comparable to transfection efficiency of Turbofect/anti-GFP siRNA complexes which showed gfp silencing expression to 35%. We did not find any differences in transfection efficiency between 2 h and 4 h of incubation regarding the GFP protein level for both siRNA concentrations. Thus, lower incubation time was used in further transfection studies and functional tests.

### 2.2. Evaluation of siRNA-Complexes Cytotoxicity

**Figure 2:**
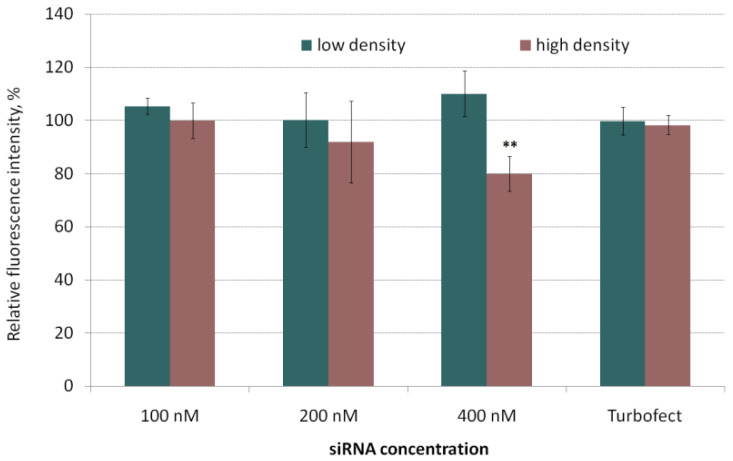
Cytotoxicity of the siRNA-polyplexes at N/P ratio 8/1 in MDA-MB-231 cells seeded with low and high density. ** *p* < 0.01 when compared with intact cells.

Cytotoxicity of siRNA-complexes is one of the important factors that must be taken into account when developing nucleic acid delivery applications. It was reported that high positive charge density of polyplexes correlates with the lower cell viability [45]. L1/siRNA polyplexes were previously shown to have positive zeta potential [39]. For cytotoxicity studies MDA-MB-231 cells were seeded with high and low densities in accordance with the cell migration and proliferation protocols. The increase in length of transfection time is considered to affect a rise of cytotoxicity [46]. To this extent, we defined the cell viability after 2-h incubation with polyplexes at different siRNA concentration (100, 200 and 400 nM) (Figure 2).

In the most cases polyplexes added to cells showed no cytotoxicity. A slight decrease in the cell viability was observed at siRNA concentration of 400 nM for cells seeded at a high density. However, the relative number of the living cells remained at 80% compared to intact cells. Thus, the siRNA polyplexes used showed no serious damaging effects on the MDA-MB-231 cells and turned out to be suitable for in vitro studies aimed at specific suppression of cell migration and proliferation.

### 2.3. RNAi-Mediated Knockdown of TNBC-Related Genes

**Figure 3:**
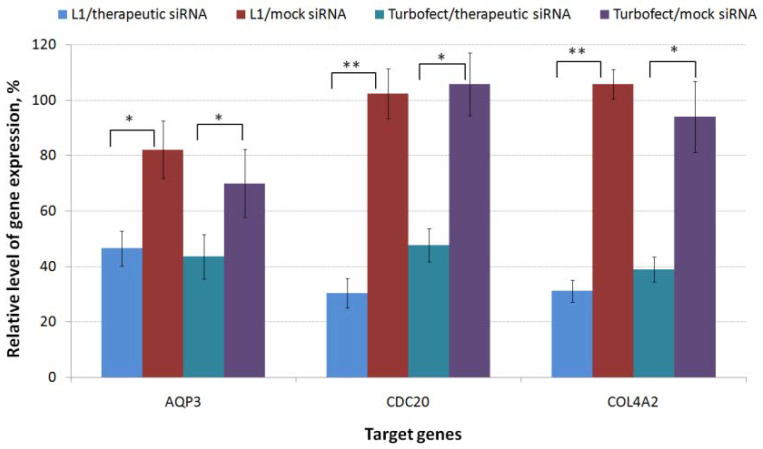
Specific silencing of gene expression after MDA-MB-231 cells treatment by polyplexes formed with L1 and anti-AQP3, anti-CDC20, anti-COL4A2 siRNA or mock siRNA. * *p* < 0.05, ** *p* < 0.01 when compared with cells treated with mock siRNA-complexes.

The lack of effective and readily available therapies that affect the proliferation and migration of TNBC cells stimulates the search for new targets and using of a combinatorial approach for targeting already known ones [47]. *AQP3*, *CDC20* and *COL4A2* genes involved in the tumor development and progression were chosen for analysis [22,27,33]. Before carrying out functional tests, antiAQP3, antiCDC20 and antiCOL4A2 siRNAs were used to confirm the specific silencing of the appropriate genes. The suppression efficacy was compared to the gene expression level after delivery of mock siRNA. siRNAs used in the experiments had a 200 nM concentration and formed polyplexes with L1 carrier at 8/1 N/P ratio. As shown in Figure 3, mock siRNA-complexes did not induce silencing of the target gene expression that confirms specificity of silencing mediated by experimental siRNAs. The cells treatment with L1/antiAQP3 siRNA complexes resulted in decrease of *AQP3* expression to 47%. L1/antiCOL4A2 siRNA polyplexes was found to decrease COL4A2 expression to 32%. The most efficient gene suppression was shown using L1/antiCDC20 siRNA polyplexes which demonstrated *CDC20* gene knockdown to 30%. The efficiency of L1/targeted siRNA polyplexes was comparable to or exceeded the efficacy of Turbofect/siRNA complexes as in the case of antiCDC20 siRNA. The results obtained suggested that the silencing of targeted genes in TNBC MDA-MB-231 cells was due to a specific RNAi but not from polyplex cytotoxicity.

### 2.4. Inhibition of MDA-MB-231 Cells Proliferation

**Figure 4:**
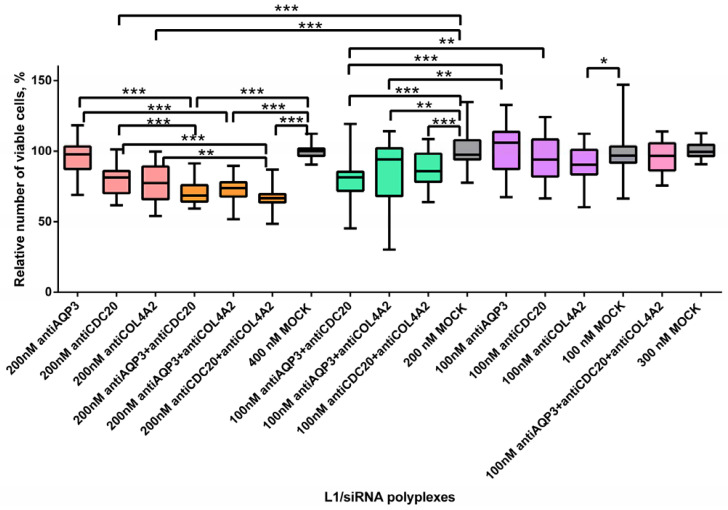
Relative number of MDA-MB-231 cells after treatment with L1 formed with therapeutic siRNAs alone and in combinations. * *p* < 0.05, ** *p* < 0.01, *** *p* < 0.001 when compared with cells treated by mock siRNA. Coloring: single siRNA polyplexes at 100 nM (purple), at 200 nM (peach); dual siRNA polyplexes at 100 nM (green), at 200 nM (orange); triple siRNA polyplexes at 100 nM (pink); mock siRNA polyplexes at 100, 200, 300 and 400 nM (grey).

The level of cell proliferative activity is known to determine the aggressiveness and malignancy of the tumor process [48]. AntiAQP3, antiCDC20 and antiCOL4A2 siRNA-polyplexes alone or in combination with each other were studied for their ability to inhibit MDA-MB-231 cell proliferation (Figure 4). Non-targeted mock siRNA-polyplexes were taken to demonstrate the cell growth suppression specificity by RNAi. siRNAs were used at 100 nM and 200 nM concentrations. We did not find significant differences between the antiAQP3 siRNA-polyplexes and mock siRNA-complexes (Figure 4). Previously, in several studies performed on different cancer cells involvement of *AQP3* gene expression in cell proliferation was demonstrated [49,50]. Arif and colleagues have demonstrated a 28% reduction in amount of MDA-MB-231 cells for stably expressing *AQP3* shRNA clone [22]. The efficiency of proliferation inhibiting may correlate with different factors such as stability and efficacy of the gene expression silencing, siRNA concentration, etc.

The results obtained demonstrated significant difference in relative cell number after their treatment with L1/antiCDC20 siRNA polyplexes at 200 nM concentration of siRNA and mock siRNA complexes. A 20% decrease in the proliferative activity of cells was found (Figure 4). Actually, silencing of *CDC20* was previously shown to inhibit MDA-MB-231 cell proliferation [51]. *CDC20* gene definitely plays a key role during mitosis of dividing cancer cells, so its silencing could decrease a tumor growth [27]. However, 100 nM concentration of antiCDC20 siRNA was not enough to decrease the cell proliferation level. The most efficient inhibition of MDA-MB-231 cell proliferation was shown using L1/antiCOL4A2 siRNA polyplexes which resulted in decrease in the cell growth by 23% at siRNA concentration of 200 nM (Figure 4). Song and colleagues using the integrated analysis showed that Col4A2 protein was the most significantly up-regulated in MDA-MB-231 cell line. Silencing of the gene resulted in inhibition in the TNBC cell proliferation [34]. Authors suppose that antiCOL4A2 siRNA treatment resulted in decrease of cell proliferation due to induction of apoptosis. Further improvement in therapeutic RNAi efficiency in TNBC cells is possible by means of alternate CXCR4-targeted siRNA delivery systems recently developed for cancer gene therapy [52,53].

To identify the synergistic effects of combinatorial siRNA delivery, several siRNAs were transfected using L1 carrier (Figure 4). AntiAQP3, antiCDC20 and antiCOL4A2 were mixed in equimolar concentrations of 100 nM and 200 nM. We also used triple siRNA-polyplexes consisted of L1 and each siRNA at the concentration of 100 nM. These polyplexes’ efficiency was compared with the efficacy of mock siRNA and each siRNA complexed alone by L1 carrier (Figure 4). The dual siRNA-polyplexes at both concentrations suppressed MDA-MB-321 cell proliferation as compared to appropriate L1/mock siRNA complexes. Moreover, complexes with a higher siRNA concentration suppressed the proliferation more efficiently (Figure 4). On the over hand, triple polyplexes with siRNA concentration of 100 nM did not decrease MDA-MB-231 cell number. We assume that several factors influencing cell proliferation could exist as the efficiency of siRNAs for gene silencing, siRNA concentration, and the amount of AGO2. An increase in the number of siRNA could lead to a self-inhibition effect caused by AGO2 depletion. Previously it was shown that a multi-knockdown siRNA system may not always effectively compete with cellular miRNAs for the AGO2 complex thus reducing RNAi efficacy [54]. Alternate combinatorial approach is an encapsulation of siRNA and chemotherapeutic drug in a single delivery system, however, this way seems very difficult due to difference in their physicochemical properties [11]. For instance, Su et al. reported a photothermal system for co-delivery of indocyanine green, paclitaxel, and survivin siRNAto TNBC cells [55]. The combined treatment resulted in significant improvement in antitumor efficacy, which opens perspectives for combinatorial application of other TNBC-related siRNAs including those described here.

We compared the efficacy of dual polyplexes with single ones formed at the same concentrations. As it can be seen in Figure 4 L1/antiAQP3 siRNA complexes did not decrease cell proliferation, whereas the addition of antiCDC20 or antiCOL4A2 resulted in a significant cell growth inhibition. The treatment by antiAQP3 and antiCDC20 or antiCOL4A2 siRNAs led to the decrease in the relative cell number to 70% or 74% at siRNA concentration 200 nM and to 81% or 82% at the concentration 100 nM, respectively (Figure 4). When compared to L1/antiCDC20 siRNA polyplexes, the addition of antiAQP3 promoted a decrease in the cell proliferation level by 10% and 15% at 200 nM and 100 nM of siRNAs, accordingly (Figure 4). Thus, due to the combinatorial targeting, we managed to demonstrate the antiAQP3 siRNA contribution to cell proliferation suppression. The combinatorial treatment with anti-CDC20 and antiCOL4A2 siRNAs-polyplexes resulted in significant inhibition of cell proliferation as compared to L1/antiCDC20 siRNA complexes only at siRNA concentration of 200 nM. However, this siRNAs combination was the most successful and led to a decrease in the cell proliferation intensity by 11% compared with anti-CDC20 siRNA only to a level of 66% (Figure 4). As compared to L1/antiCOL4A2 siRNA complexes significant differences were registered only for combinatorial treatment by antiCDC20 and antiCOL4A2 siRNAs at 200 nM of siRNA concentration. To sum up, the combinatorial delivery of antiCDC20 and antiCOL4A2 siRNAs seems to be more effective for the inhibition of cell growth.

### 2.5. Inhibition of MDA-MB-231 Cells Migration

**Figure 5:**
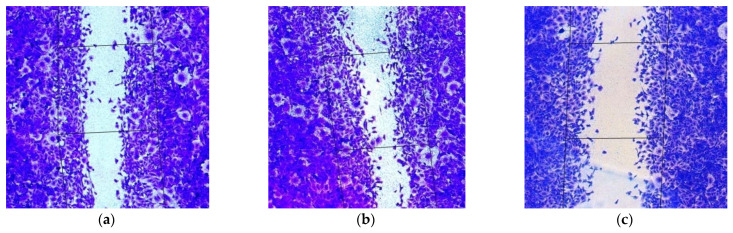

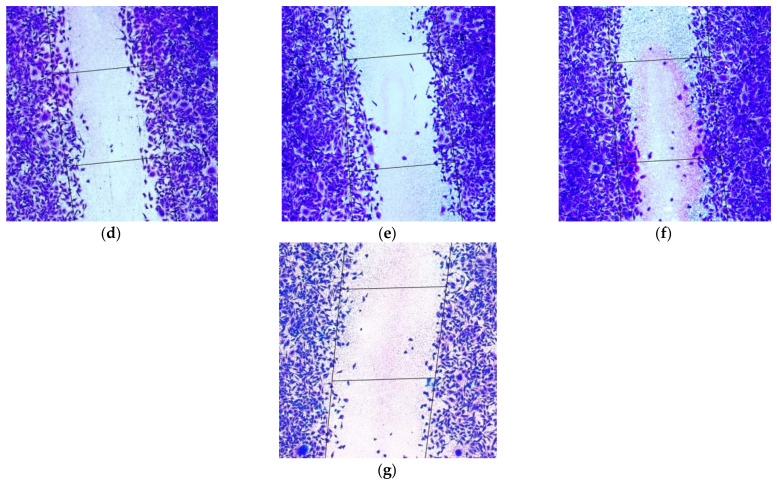
Appearance of migrated MDA-MB-231 intact cells (magnification ×100) (**a**) and after their treatment with (**b**) L1/mock siRNA (200 nM), (**c**) L1/mock siRNA (400 nM), (**d**) L1/anti-AQP3 siRNA (200 nM), (**e**) L1/anti-CDC20 siRNA (200 nM), (**f**) L1/anti-COL4A2 siRNA (200 nM) and (**g**) L1/anti-CDC20 siRNA+anti-COL4A2 siRNA (200 + 200 nM) complexes.

Active migration of cancer cells is a prerequisite for their invasion and metastasis [56]. A scratch assay was performed to determine whether MDA-MB-231 cell migration was affected by antiAQP3, antiCDC20, or antiCOL4A2 siRNAs as well as their dual and triple combinations. L1/mock siRNA polyplexes were used as a negative control and to demonstrate the specificity of RNAi functional effects. MDA-MB-231 cells were treated with L1/siRNA-polyplexes with different siRNA concentrations. The cell monolayer was damaged and the number of cells that migrated into the cell-free area was counted; the number of migrated untreated cells was taken as 100% (Figure 5).

**Figure 6:**
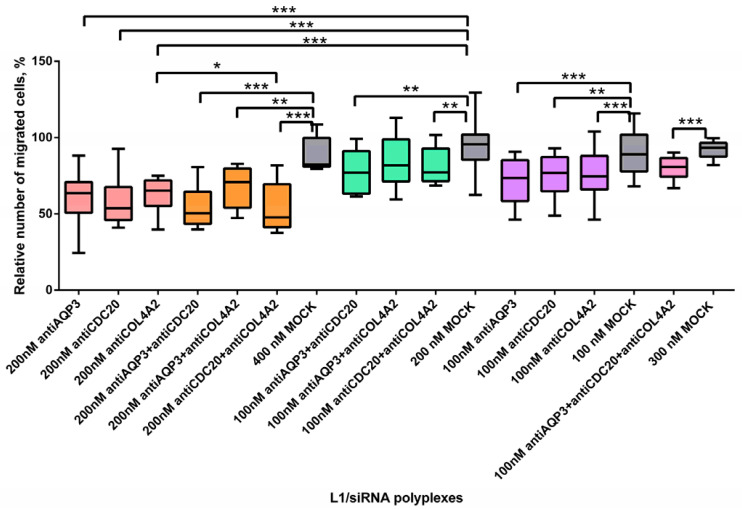
Relative number of migrated MDA-MB-231 cells (%) after treatment with L1 formed with therapeutic siRNAs aloneand in combinations. * *p* < 0.05, ** *p* < 0.01, *** *p* < 0.001 when compared with cells treated by mock siRNA. Coloring: single siRNA polyplexes at 100 nM (purple), at 200 nM (peach); dual siRNA polyplexes at 100 nM (green), at 200 nM (orange); triple siRNA polyplexes at 100 nM (pink); mock siRNA polyplexes at 100, 200, 300 and 400 nM (grey).

Previous results suggested that *AQP3* is one of the key regulators of MDA-MB-231 migration [22]. After L1/antiAQP3 siRNA polyplexes cell treatment decrease of cell migration level was found to be to 73% and 63% with 100 nM and 200 nM of siRNA, respectively, in comparison with corresponding mock siRNA-complexes (Figure 6). Satooka and Hara-Chikuma demonstrated the importance of AQP3 for SDF-1/CXCR4-dependant migration of the breast cancer cells [20]. Moreover, *AQP3* was shown to be a necessary component for fibroblasts growth factor 2-induced cell migration [57].

Aberrant *CDC20* expression could play a role in the aggressive metastasizing of TNBC [26]. Under antiCDC20 siRNA-treatment, MDA-MB-231 cells showed a significantly reduced level of migration to 77% and 54% at siRNA concentrations of 100 nM and 200 nM, respectively, as compared to L1/mock siRNA complexes (Figure 6). In fact, *CDC20* is one of the main factors for cancer cell migration and its overexpression promotes the metastasizing ability of the breast cancer cells [58]. CDC20 overexpression may be associated with an aggressive course of TNBC [26]. Abnormal *CDC20* expression leads to mitotic errors which are the main reasons for chromosome instability (CIN). Bakhoum and colleagues demonstrated that CIN facilitated the cell metastasis through cGAS–STING-dependent manner (cyclic GMP-AMP synthase–stimulator of interferon genes) [59]. Paul and colleagues previously showed CDC20-mediated proteasomal degradation of SMAR1 (the tumor suppressor scaffold matrix attachment region binding protein 1) facilitated the cancer cell migration [60].

For L1/antiCOL4A2 siRNA polyplexes we also demonstrated significant differences in the MDA-MB-231 cell migration efficacy compared to mock siRNA-complexes. The treatment with antiCOL4A2 siRNA-polyplexes promoted a decrease in relative migration level to 75% and 65% (Figure 6). The important role of the *COL4A2* gene in TNBC cell migration was already demonstrated previously [34]. The authors suppose the *COL4A2* gene targets the Jak-STAT signaling pathway, which in turn is one of the TNBC-specific regulatory feedback programs [34,61].

Thus, treatment of TNBC MDA-MB-231 cells with antiAQP3, antiCDC20, or antiCOL4A2 siRNA-polyplexes resulted in strongly reduced cell migration ability. The combinatorial siRNAs delivery can allow us to determine more effective therapeutic combinations to prevent breast cancer metastasis.

We found that using triple siRNAs-polyplexes resulted in a significant decrease of the cell migration rate in comparison with L1/mock siRNA complexes (Figure 6). The level of MDA-MB-231 cell migration dropped to 80%. However, no significant difference was found when compared with single siRNA-polyplexes treatment at 100 nM of concentration. Most of dual polyplexes at both concentrations effectively suppressed MDA-MB-321 cell migration as compared to corresponding mock siRNA complexes (Figure 6). Significant increase of cell migration was demonstrated when using antiCDC20 and antiCOL4A2 siRNAs in comparison with single antiCOL4A2 siRNA treatment. Transfection with antiCDC20 siRNA and antiCOL4A2 siRNA-polyplexes facilitated a decrease in the cell migration level to 48% at 200 nM of each siRNA (Figure 6). It can be suggested that combinatorial delivery in most cases did not lead to a cumulative effect due to significantly reduced level of the cell migration caused by single siRNA-polyplexes (Figure 6).

The data obtained are summarized in Table 1. The most profound synergistic anti-proliferative and migration inhibiting activity was found for co-delivery of antiCDC20 and antiCOL4A2 siRNAs. In other cases we demonstrated that silencing of several targets is not always beneficial for effective anti-cancer therapy. However, the positive results of the previous study on combinatorial delivery of antiCD20 and anti-phosphatase siRNAs confirm our findings on revealed synergistic effects [27]. Thus, it can be suggested that combinatorial siRNA delivery against *CDC20* and *COL4A2* could be a promising strategy for RNAi-based TNBC therapy.

## 3. Materials and Methods

### 3.1. Cell Lines

GFP-positive triple negative breast cancer cells MDA-MB 231 were cultivated under mycoplasma-free conditions in DMEM (Biolot LLC, Saint Petersburg, Russia) supplemented with 10% St-Biol—heat-inactivated fetal calf serum (Biolot LLC), 2 mM L-glutamine with addition of 0.012% of gentamicin as described previously [62].

### 3.2. Peptide Synthesis and Design

L1 peptide was synthesized by NPF Verta, LLC (Saint Petersburg, Russia) using solid-phase Boc-chemistry as described previously [41]. A dry powder of the peptide was stored at −70 °C. A dry sample (1–2 mg) was dissolved in 0.1% TFA at 2 mg/mL and stored at −20 °C. A high-performance liquid chromatography was used to determine the peptide purity which was in the range 90–95%. L1 peptide consists of the KPVSLSYRSPSRFFESH ligand part linked with DNA-binding motif (CHRRRRRRHC) by two ε-aminocaproic acids (Ahx) [38].

### 3.3. siRNA, Preparation of Peptide/siRNA Complexes

The sense sequence of anti-AQP3 siRNA 5′-GGG UCG UCA CUC CUU UAA Utt-3′ targets human *AQP3* mRNA [63]. The sense sequence of anti-CDC20 siRNA 5′-GGG AAU AUA UAU CCU CUG Utt-3′ targets human *CDC20* mRNA [64]. The sense sequence of anti-COL4A2 siRNA 5′-GGC AGA AAG GUG AGC CUU Att-3′ targets human *COL4A2* mRNA [65]. The sense sequence of anti-GFP siRNA 5′-CAA GCU GAC CCU GAA GUU Ctt-3′ targets *gfp* mRNA [66]. A non-silencing siRNA 5′-UUC UCC GAA CGU GUC ACG U-3′ served as a mock siRNA [67]. siRNAs were synthesized by Syntol JSC, Moscow, Russia. The peptide/siRNA complexes were prepared at 8 to 1 peptide nitrogen/RNA phosphorus ratio (N/P ratios). siRNA solution was obtained by dilution of required amount of siRNA to 100 μg/m Lin Hepes-buffered mannitol (HBM) (5% *w*/*v* mannitol, 5 mM Hepes, pH 7.5). The L1 peptide at a certain N/P ration diluted in HBM was added to the siRNA solution in equal volume and mixed. Complexes were kept at room temperature for 30 min.

### 3.4. Cytotoxicity Assay

Cytotoxicity of peptide/siRNA complexes added to cells with low and high density was evaluated using Alamar blue assay (BioSource Intl., Camarillo, CA, USA). 0.6 × 10^4^ (low density) and 2 × 10^4^ (high density) of MDA-MB-231 cells were seeded in 96-well plates a day before experiment. The cytotoxicity experiments were carried out as described previously [38]. The cell viability was studied after 16 h of cell incubation with AlamarBlue. The fluorescence was measured on a Wallac 1420D scanning multilabel counter (Thermo Fisher Scientific Oyj, Vantaa, Finland) using excitation and emission wavelength at 544 nm and at 590 nm, consequently. Relative fluorescence intensity was counted by (F − Ff)/(Fb − Ff) ∗ 100%, where Fb is a fluorescence intensity in untreated control and Ff is a fluorescence intensity without cells. The results are presented as mean ± S.E.M from three independent experiments with three samples.

### 3.5. siRNA Transfer to MDA-MB-231 Cells

5.2 × 10^4^ or 10.5 × 10^4^ cells at high density were seeded in 48-well or 24-well plates, respectively, and incubated overnight. Before transfection the medium was replaced with serum-free one.Anti-GFP siRNA and mock siRNA-containing polyplexes were added in 48-well plates and incubated with cells for 2 and 4 h. L1 complexed with anti-AQP3, anti-CDC20, anti-COL4A2 and mock siRNAs were added in 24-well plates and incubated with cells for 2 h. The final concentrations of siRNAs were 100 or 200 nM and volumes of medium were 500 and 1000 µL for 48- and 24-well plates, accordingly. After incubation in full culture medium for the next 48 h, cells from 48-well plates were permeabilized for further GFP fluorescence measurement with a Wallac 1420D scanning multilabel counter (Thermo Fisher Scientific Oyj) at 485 nm excitation and 535 nm emission wavelengths as described previously [41]. The GFP fluorescence was normalized by the total protein concentration, measured with Bradford reagent (Helicon, Moscow, Russia). The results are presented as mean ± S.E.M from three independent experiments with three samples. After 48 h of incubation cells from 24-well plates were prepared for RNA extraction with Trizol reagent (Qiagen, Düsseldorf, Germany) and subsequent cDNA synthesis using an OT-1 Reverse Transcription kit (Syntol JSC) according to the manufacturer’s instructions.

### 3.6. Quantitative RT-PCR

Quantitative PCRs were carried out with EvaGreen PCR kit (Syntol JSC) using *AQP3* forward primer 5′-GCAGCCTGTCCATCTGTG-3′, reverse primer 5′-ACCCTACTTCCCAAAAGCC-3′; *CDC20* forward primer 5′-CGCTATATCCCCCATCGCAG-3′, reverse primer 5′-GATGTTCCTTCTTGGTGGGC-3′; *COL4A2* forward primer 5′-ATTCCTTCCTCATGCACACG-3′, reverse primer 5′-ACTTGTTGGCGTAGTAGTGG-3′, and β-actin gene as endogenous reference was detected using forward 5′-TGCCGACAGGATGCAGAAG-3′, reverse primer 5′-GCCGATCCACACGGAGTACT-3′. 1.25 µg of cDNA was added to the reaction [39,63,64,65]. As previously, PCR was performed in Rotor-Gene 3000 thermocycler (Corbett Life Science, Sydney, Australia) with the following conditions:94 °C for 10 min, followed by 40 cycles of 95 °C for 20 s and 60 °C for 1 min; subsequent qPCR analysis was carried out using the Rotor-Gene version 6.1.71 software [39]. A standard curve of Ct value vs. log amount of standard was generated for each gene using 5-times dilutions of cDNA (1.6; 0.8; 0.4; 0.2; 0.1 µg) prepared from untreated cells. The values are presented as mean ± S.E.M from three independent experiments with two samples.

### 3.7. Proliferation Assay

0.6 × 10^4^ of MDA-MB-231 cells were seeded in a 96-well plate a day before experiments. Cell culture medium was replaced with 50 µL of serum-free medium. L1 complexed with siRNAs in concentrations of 100, 200 and 300 nM were added and incubated with cells for 2 h. Then cell culture medium was replaced with 100 µL of medium containing 2.5% FBS for the next 72 h for further cell proliferation to be analyzed using Alamar blue assay [41]. The fluorescence was measured and relative fluorescence intensity was counted as described above (see Section 3.4). The results are shown as mean ± S.E.M from five independent experiments with four samples.

### 3.8. Scratch Migration Assay

2 × 10^4^ of MDA-MB-231 cells were seeded into a 96-well plate 24 h before transfection. Transfection was performed as described above (see Section 3.5) in 175 mkl of serum-free medium. The final concentrations of siRNAs were 100, 200 and 300 nM. After 2 h incubation with L1/siRNA polyplexes, cells were washed by medium, the monolayer was scratched with a 300 μL pipette tip (Biohit Oyj, Helsinki, Finland) with subsequent cell wash by Hanks’ solution and photographing of wound area as described previously [39]. After cell incubation in FBS-containing medium (10%) for the 24 h cells were washed, stained in 100 µL of 0.2% crystal violet in 5% ethanol, dried at 37 °C overnight and photographed by MIBR microscope (LOMO, Saint Petersburg, Russia). Number of cells (N) migrated to wound area was counted using ImagePro Plus 6.0 software (Media Cybernetics, Rockville, MD, USA). Relative number of migrated cells was calculated by (N/N′) ∗ (ρ′/ρ), where N′ is number of migrated cells in untreated control and ρ′ is cell density in untreated control. The results are presented as mean ± S.E.M from five independent experiments with four samples.

### 3.9. Statistical Analysis

Statistical analysis was performed by Student’s *t*-test using GraphPad Prism5 Software (GraphPad, McIntosh, CA, USA). Statistical significance was defined as * *p* < 0.05, ** *p* < 0.01 and *** *p* < 0.001.

## 4. Conclusions

Targeting of triple negative breast cancer-related genes by means of RNA interference is a very promising approach for treatment of this deadly disease. It should be noted that an efficient and TNBC-specific siRNA delivery system is a prerequisite for the development of RNAi-based medicines. The efficiency of an anti-proliferative treatment can be further improved via the combinatorial delivery of siRNAs against multiple targets responsible for proliferative activity in TNBC cells. Here, we have demonstrated the efficient down-regulation of TNBC cells migration and proliferation by antiAQP3, antiCDC20, and antiCOL4A2 siRNA delivery mediated by peptide-based vector L1. Several types of L1-based siRNA polyplexes have been tested and the most efficient combination of siRNAs has been found. Particularly, co-delivery of antiCDC20 and antiCOL4A2 siRNAs synergistically inhibited migration and proliferation of MDA-MB 231 cells. Therefore, the combinatorial strategy based on co-delivery of antiCDC20 and antiCOL4A2 siRNAs by means of CXCR4-targeted non-viral vehicle could be a promising approach to further development of RNAi-based therapy of TNBC.

## Figures and Tables

**Table 1:** Summary of registered anti-proliferative effects after antiAQP3, antiCDC20, or antiCOL4A2 siRNA delivery and co-delivery mediated by an L1 carrier (↓ means decrease of migration/proliferation; - means no effect; ↓↓ means synergistic effect; ↓↓↓ means the most profound synergistic effect).

Type of Analysis	Type of siRNA					
	AntiAQP3	AntiCDC20	AntiCOL4A2	AntiAQP3 + AntiCDC20	AntiAQP3 + AntiCOL4A2	AntiCDC20 + AntiCOL4A2
Proliferation	-	↓	↓	↓↓	↓↓	↓↓↓
Migration	↓	↓	↓	↓	↓	↓↓

## Data Availability

The data presented in this study are available on request from the corresponding author. Authors can confirm that all relevant data are included in the article.
